# Recirculation of *Giardia lamblia* Assemblage A After Metronidazole Treatment in an Area With Assemblages A, B, and E Sympatric Circulation

**DOI:** 10.3389/fmicb.2020.571104

**Published:** 2020-10-22

**Authors:** Maria Fantinatti, Luiz Antonio Pimentel Lopes-Oliveira, Tiara Cascais-Figueredo, Phelipe Austriaco-Teixeira, Erika Verissimo, Alexandre Ribeiro Bello, Alda Maria Da-Cruz

**Affiliations:** ^1^Laboratório Interdisciplinar de Pesquisas Médicas, Instituto Oswaldo Cruz, Fundação Oswaldo Cruz, Rio de Janeiro, Brazil; ^2^Disciplina de Parasitologia, Faculdade de Ciências Médicas, Universidade do Estado do Rio de Janeiro, Rio de Janeiro, Brazil

**Keywords:** *Giardia lamblia*, assemblage, metronidazole, reinfection, parasite persistence, resistance

## Abstract

*Giardia lamblia* is an intestinal protozoan subdivided into eight assemblages, labeled alphabetically from A to H. Assemblages A, B, and E infect humans and can have a sympatric circulation. We investigated the assemblage recirculation in children living within a high prevalence area of *Giardia* infection. One hundred and ninety-four children were evaluated and 85 tested positive for *Giardia* by PCR. These infected individuals were recruited, treated with metronidazole and then reexamined for infections at 20 and 40 days after treatment that included PCR and the genotyping was performed by sequencing beta-giardin and glutamate dehydrogenase gene targets. *Giardia* assemblages A (*n* = 43), B (*n* = 21), E (*n* = 17), and A/E (*n* = 4) were identified in infected children. Assemblage A was found in all reoccurrences of infection, including four that had been infected by assemblages B and E. Since both persistence and reinfection could account for the results, the level of nucleotide homology was determined before and after treatment. Most suggested that reinfections were by the same strain, but four presented a distinct genotypic profile. The results suggest that the differences in the genotypic profiles were due to reinfections, which appear to occur with frequency in high *Giardia* burden areas and soon after the end of therapy. It is not yet possible to define whether the recurrent cases were related to parasite resistance. However, the evidence of rapid reinfections and ready availability of treatment raises the potential for creating resistant strains. This highlights the need to address how *Giardia* burden is maintained within high prevalence areas.

## Introduction

*Giardia lamblia* is a flagellated protozoan that infects the small intestine of a broad spectrum of mammalian hosts. It is transmitted by a fecal-oral route that is closely associated with poor sanitary conditions. Recent surveys estimate that over 183 million individuals infected by *Giardia* in the world (Torgerson et al., 2015).

Phylogenetically, *G. lamblia* is divided into eight assemblages labeled alphabetically from A to H. Humans are mainly infected by assemblages A and B (Adam, 2001; Feng and Xiao, 2011; Cacciò et al., 2018). The presence of assemblage E has also been detected although at a much lower frequency than A and B (Fantinatti et al., 2016; Scalia et al., 2016; Zahedi et al., 2017). The specific characteristics of each of the different assemblages raises the possibilities for unique factors associated with virulence, pathogenesis and drug susceptibility. However, as an infection is often asymptomatic, the diagnosis and treatment of infected individuals can be difficult.

In Brazil, the main class of drugs recommended for the treatment of giardiasis is 5-nitroimidazole. Despite their effectiveness, 5-nitroimidazole drugs share a number of side effects (Gardner and Hill, 2001). The most commonly used drug is metronidazole due to its low cost and easy access. Although treatment with metronidazole appears to be effective in a majority of infections, there is emerging evidence for an increased frequency of therapeutic failure (Tejman-Yarden and Eckmann, 2011; Leitsch, 2015). In London, United Kingdom, an exorbitant increase in persistent parasitic infection following 5-nitroimidazole treatment was reported covering the period 2008 to 2013 where therapeutic failure increased from 15.1 to 40.2% (Nabarro et al., 2015). A number of reasons can be attributed to this apparent increase such as inadequate dosages, incomplete treatment regimens, immunosuppression or drug resistance (Lemée et al., 2000; Gardner and Hill, 2001; Nash et al., 2001; Escobedo et al., 2014).

In Brazil, *Giardia* infection is spreaded over the country and the estimated point prevalences may reach over 50% in areas of social and sanitary vulnerability (Coelho et al., 2017). It points to that transmission rate of the parasite is high. On the other hand, to date, the occurrence of persistent infection is unknown. We hypothesize that the biological characteristics of each of the circulating assemblages could influence their detected incidence and drug susceptibility. The current study was undertaken to investigate the circulation of *G. lamblia* assemblages in an area with a high prevalence of infection after treatment of parasitized individuals with metronidazole.

## Materials and Methods

### Sample Collection, Parasite Diagnosis, and Treatment

The study was performed on a daycare center located within an economically challenged community of Rio de Janeiro, Brazil. A total of 194 children in this day care center of ages from 10 months to 4 years old were evaluated for 2 years. A child was only included in the research after a parent accepted an invitation to participate signed a Term of Free and Informed Consent, which was approved by an Institutional ethical review board (number CAAE:19705613.9.0000.5248) and included full disclosure of benefits and risks. A single initial stool sample was collected from each patient for the diagnosis of geohelminths and protozoa. The samples were submitted to parasitological examination of feces by Ritchie and Kato-katz techniques. Additionally, a molecular diagnosis by PCR was performed to identify the presence of *Giardia* DNA (see below).

Individuals with a positive diagnosis for pathogenic parasites were treated according to guidelines of the Brazilian ministry of health. For *Giardia* infections, patients were given metronidazole 15 mg/kg/day orally (maximum 250 mg) for 5 or 7 days in cases of treatment failure. Around 20–35 days after treatment, a new sample was collected to define cure control. Individuals who remained positive were submitted to a second cycle of metronidazole treatment and then reevaluated after 20 days (second cure control). The diagnostic methodology of the samples of the cured controls were the same used in the first examination.

### DNA Extraction and Molecular Characterization of *G. lamblia*

Samples were stored at −20°C until molecular diagnosis and characterization. DNA extraction from cysts was performed directly from the stool using the QIAamp DNA Stool mini kit (Qiagen, Germany), mostly according to the manufacturer’s instructions. Two exceptions were lysis temperature, which was increased to 95°C, and the volume of AE buffer used for DNA elution, which was decreased to 100 μL. Isolated DNA was stored at −20°C until the time of use.

For genotyping of *G. lamblia*, regions of the conserved genes coding for glutamate dehydrogenase (*gdh*) and Beta-giardin (β*-gia*) were utilized. Extracted DNA was submitted to PCR and nested PCR (secondary PCR), in a final volume of 50 μL of reaction containing 1X PCR buffer, 3 mM of MgCl_2_, 2.5 U of Taq DNA Polymerase (Invitrogen Life Technologies, Brazil), 200 μM of triphosphate deoxyribonucleotides dNTP (Invitrogen Life Technologies, Brazil) and 0,2 μM of each primer.

For amplification of the *gdh* target, primers were used in primary PCR: GDH 1 (TTCCGTRTYCAGTACAACTC) and GDH 2 (ACCTCGTTCTGRGTGGCGCA) and in secondary PCR: GDH3 (ATGACYGAGCTYCAGAGGCACGT) and GDH4 (GTGGCGCARGGCATGATGCA), according to Cacciò et al., 2008. For amplification of the β*-gia* target, primers were used in primary PCR: G7 (AAGCCCGACGACCTCACCCGCAGTGC) and G759 (GAGGCCGCCCTGGATCTTCGAGACGAC) and in secondary PCR: B-GIAF (GAACGAACGAGATCGAGGTCCG) e B-GIAR (CTCGACGAGCTTCGTGTT), according to Cacciò et al., 2002 and Lalle et al., 2005, respectively.

As a positive control, DNA extracts from axenic cultures of the C6 clone of the WB strain of *G. lamblia* (ATCC 50803) were used. Negative controls included DNA extracted from axenic cultures of *Entameba histolytica* and *Trichomonas vaginalis*. Successful amplification was verified by agarose gel electrophoresis at 1% concentration.

PCR products for each pair of primers were purified using the NucleoSpin Gel and PCR Clean-up kit (Macherey-Nagel, Germany) according to manufacturer’s instructions with the exception of incubation time in NE buffer, which was raised to 5 min. The purified products were subjected to sequencing in both directions in triplicate using the BigDye Terminator v3.1 Cycle Sequencing Kit (Applied Biosystems, Foster City, United States). Following precipitation of the reaction, samples were analyzed at the DNA Sequencing Platform sequencing service of FIOCRUZ (Otto et al., 2007). Each experiment was performed in triplicate.

Resulting electropherograms were analyzed for quality in the program Chromas 2.4. Sequence characterization was performed using the Basic Local Alignment Search Tool using nucleotide (BLASTn) and the consensus was obtained by the CAP3 Sequence Assembly^1^ Program. The nucleotide sequences of *gdh* and β*-gia* from *G. lamblia* were aligned by the algorithm CLUSTAL W (Thompson et al., 1994) in the Molecular Evolutionary Genetics Analysis (MEGA) 7.0 package (Kumar et al., 2016).

The phylogenetic analysis was performed using the MEGA 7.0 program and the distance estimation used was based on the equation by Jin and Nei (1990) using Kimura model 2-parameters). Sequences of novel isolates in clinical samples were aligned using GenBank *G. lamblia* sequences belonging to assemblages A, B, and E. As outgroups, sequences from *G. psittaci*, *G. muris*, and *T. vaginalis* were used. The respective accession numbers of the sequences are between vertical bars in the phylogenetic trees. Additionally, the sequences of the *gdh* and β*-gia* gene fragments were concatenated and the bootstrap above 60% was reported.

Phylogenetic trees were constructed using the neighbor-joining algorithm (Saitou and Nei, 1987). The best statistical model was defined from the lowest BIC value found in the MEGA (Kumar et al., 2016), where Kimura 2-parameter was chosen for both genes. For each construct, the veracity of each branch was conferred by the confidence limit by bootstrap analysis (1000 repetitions) (Felsenstein, 1985). The characteristic of *G. lamblia* sequence obtained from GenBank and used as a reference for the construction of *gdh* and β*-gia* genes phylogenetic tree were presented in Supplementary Table 2.

The Recombination Detection Program version 4 (RDP4) software was used to verify recombination events in our isolates from clinic samples, using the alignment of concatenated sequences for *gdh* and β*-gia* genes (Martin et al., 2015). The existence of unique events recombination was verified by multiple methods (RDP, GENECONV, BootScan, MaxiChi, Chimera, SiScan, and 3Seq).

### Evaluation of Nucleotides Sequence in *G. lamblia* Trophozoites Continuously Exposed to Metronidazole *in vitro*

*Giardia lamblia* trophozoites from W6 clone, WB strain [ATCC50803], were cultured at 37°C in TYI-S-33 medium at pH 7.0 supplemented with 10% inactivated fetal bovine serum (Cultilab, Campinas, Brazil). Trophozoites were continuously exposed to metronidazole (MTZ) (Sigma Chemical Co., St. Louis, United States) for a minimum of 16 weeks. Experimental groups were defined by MTZ exposure concentration: group 1 (MTZ5), 5 μM; group (MTZ10) 2, 10 μM; group 3 (MTZ20), 20 μM; group 4 (SMTZ), no exposure to metronidazole; and group 5 (CDMSO), exposed to 0.05% dimethylsulfoxide (Sigma, United States), the vehicle for MTZ dilution. The concentration required to inhibit 50% parasitic growth (IC_50_), was determined using the methodology described by Bénéré et al., 2007. The assays for each experimental group were performed in triplicate and the IC_50_ results were determined using Prism 6.0 software (GraphPad;^2^). The ANOVA test was used for statistical analysis.

At the end time point, *G. lamblia* trophozoite DNA was extracted using DNAzol (1 mL/10^7^ parasites; Invitrogen, Carlsbad, CA, United States). Sequences of *gdh* and β*-gia* were obtained and analyzed as described above.

## Results

### Frequency of *G. lamblia* Infection and Determination of Parasite Persistence After Treatment

**FIGURE 1:**
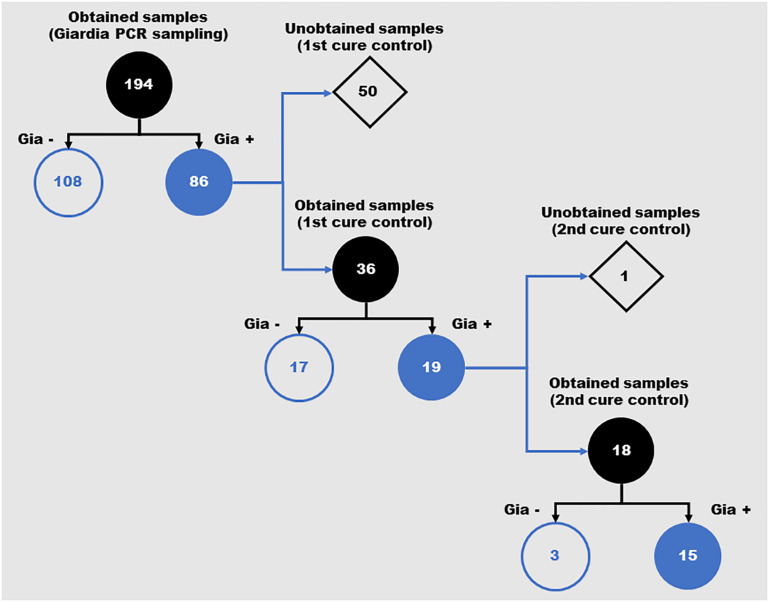
Schematic representation of the positivity for *Giardia lamblia* in feces samples from children over the different periods of the study. Number of samples obtained during the three evaluation moments of the study (*Giardia* PCR sampling, 1st cure control and 2nd cure control). Gia–: Samples with negative diagnosis for *Giardia* by PCR; Gia+: Samples with positive diagnosis for *Giardia* by PCR.

A total of 194 stool samples were collected from children, 109 females and 85 males. In the first examination, the following parasites were found: *Ascaris lumbricoides* (15/194 – 7.73%), *Entameba histolytica/dispar* (4/194 – 2.06%), *Entameba coli* (5/194 – 2.58%), *Endolimax nana* (13/194 – 6.70%) and *G. lamblia* (86/194 – 44.33%) (Supplementary Table 1). All individuals positive for *G. lamblia* were treated with metronidazole and, after treatment, 36 remained in the study group for evaluation. Nineteen cases presented as a persistent infection after treatment and underwent a second cycle of treatment with metronidazole. In the determination of the second cure control, 18 individuals were reevaluated. Of these, fifteen still presented a positive diagnosis for *G. lamblia* (Figure 1). These cases were individually monitored for other clinical approaches.

### Genotyping of *G. lamblia* Circulating in Preschoolers Before and After Treatment

**FIGURE 2:**
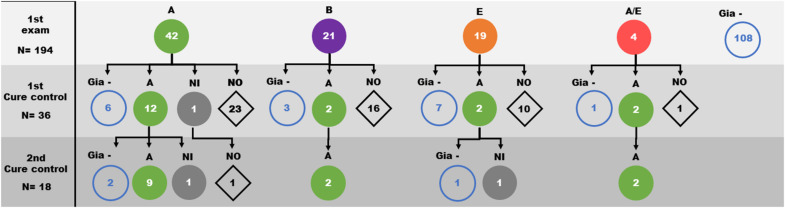
Distribution of *Giardia lamblia* assemblages (circles) identified in faeces samples from children over the three evaluation moments of the study (1st *Giardia* PCR sampling, 1st cure control and 2nd cure control). A: Assemblage A (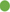); B: Assemblage B (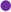); E: Assemblage E (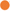); A/E: Assemblage A/E (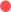); NI: Assemblage not identified (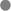); Gia– : samples with negative PCR to *Giardia* (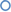); NO: sample not obtained (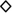).

**FIGURE 3:**
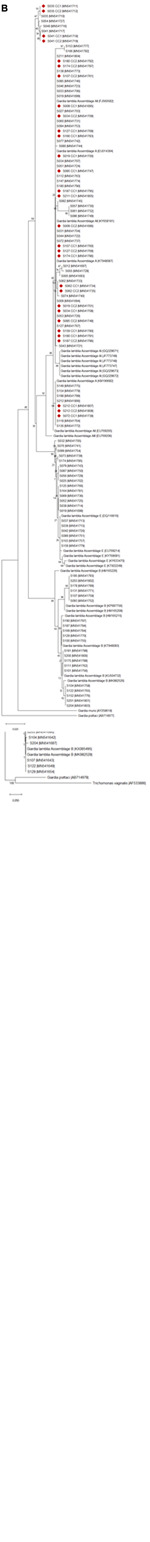
Phylogenetic tree of the *Giardia lamblia* isolates collected from children before and after treatment, by the neighbor-joining algorithm using a Kimura two-parameter. **(A)** Phylogenetic tree based on sequences of the *glutamate dehydrogenase* gene. **(B)** Phylogenetic tree based on sequences of the *beta-giardin* gene. The name sequences are indicated by their accession numbers. Red diamond: CC1: isolate from sample of first cure control, and CC2: isolate from sample of second cure control.

The *G. lamblia* DNA isolated from the 86 children positive at the initial diagnosis were genotyped using both *gdh* and β-*gia* as targets. We observed a great genetic variability among the isolates from samples. From the consensus sequences, samples were grouped as: 40 isolates in assemblage A, 21 isolates in assemblage B, 17 isolates in assemblage E and 4 isolates showing characteristics of both assemblage A and E (Figures 2, 3). Many differences were observed in subassemblages characterization AI and AII.

From the first cure control exam, 36 diagnosed cases were still positive for *Giardia* infection and were reevaluated. Based on the initial determination of the assemblage group, 19 isolates were from assemblage A, 5 isolates from assemblage B, 9 isolates from assemblage E and 3 isolates from assemblage A/E. After the second course of treatment, 13 of 19 assemblage A, 2 of 5 assemblage B, 2 of 9 assemblage E and 2 of the 3 assemblage A/E children were still positive. Genotyping showed that 18 of them were grouped as assemblage A (Table 1 and Figure 3 and Supplementary Figure 1). Curiously, the assemblages B or E were not found among positive individuals (Figure 2 and Supplementary Figure 1).

**TABLE 1:** *Giardia lamblia* assemblages found at first diagnosis and after treatment.

**Cases who realized 1st examination**	**Cases diagnosed as *Giardia* infection by PCR (number of samples)**	**Cases who realized 2nd evaluation**	**1st cure control (number of samples)**	**Cases who realized 3rd evaluation**	**2nd cure control (number of samples)**
194	PCR Negative (*n* = 108)	−	−	−	−
	
	Assemblage A (*n* = 42 cases)	19	PCR Negative (*n* = 6)	−	−
					
			Assemblage A (*n* = 12)	12	PCR Negative (*n* = 2)
					Assemblage A (*n* = 9)
					
					Not identified (*n* = 1)
			
			Not identified (*n* = 1)	−	−
	
	Assemblage B (*n* = 21 cases)	5	PCR Negative (*n* = 3)	−	−
			Assemblage A (*n* = 2)	2	Assemblage A (*n* = 2)
	
	Assemblage E (*n* = 19 cases)	9	PCR Negative (*n* = 7)	−	−
			
			Assemblage A (*n* = 2)	2	PCR Negative (*n* = 1)
					
					Not identified (*n* = 1)
	
	Assemblage A/E (*n* = 4 cases)	3	PCR Negative (*n* = 1)	−	−
					
			Assemblage A (*n* = 2)	2	Assemblage A (*n* = 2)

*−: no samples were obtained.*

Among the individuals infected by assemblage A (11 subjects) in the first evaluation and in the first cure control, 9 isolates presented 100% of identity between the isolates from samples before and after treatment for the two gene targets used (*gdh* and β*-gia*). Three individuals presented a genotypic divergence between the isolates from first evaluation analyzed and the first cure control. Two showed differences in the β*-gia* gene and other showed differences in the *gdh* target.

After the 2nd course of treatment, 15 cases continued to be positive with 13 isolates being grouped to the assemblage A, which included the identification of a unique assemblage. All sequences from second cure control showed 100% identity with the sequence from the same individual in the first cure control.

Three samples that were positive after treatment, one from the 1st cure control and two were from the 2nd cure control groups, were amplified but not characterized. Three other isolates were genotyped only based on *gdh* gene.

Among the individuals that presented isolates grouped as assemblage A in the first exam and in the successive cure controls, 8 cases showed 100% identity of the isolates at all three moments of analysis.

Using RDP4, the number of possible unique events recombination among concatenated sequences was different according to the detection method (RDP: 13, GENECONV: 15, BootScan: 9, MaxiChi: 9, Chimera: 11, SiScan: 20, 3Seq: 19). In region of β*-gia* gene, few recombination events were found (RDP: 0, GENECONV: 1, BootScan: 0, MaxiChi: 1, Chimera: 1, SiScan: 1, 3Seq: 2). The major number of recombination was observed in the *gdh* gene region (RDP: 13, GENECONV: 9, BootScan: 6, MaxiChi: 6, Chimera: 6, SiScan: 9, 3Seq: 13). Both isolates of assemblage B and assemblage E presented one recombination event. Demonstrating the same phylogenetic profile among isolates from each assemblage cluster. All other recombination events were observed in assemblage A for the gdh target.

### No Changes in Nucleotides Sequences of *gdh* and β*-gia* From *G. lamblia* Trophozoites Were Observed After *in vitro* Exposure to Metronidazole

The IC_50_ values of the groups MTZ5, MTZ10, and MTZ20 were significantly higher than those presented by the control groups SMTZ and CDMSO. No differences on the nucleotide sequences were observed among the groups (Lopes-Oliveira et al. submitted). These results demonstrated that despite a profound interference on the survival of the parasite, MTZ does not induce alterations in the conserved genes evaluated.

## Discussion

The *G. lamblia* transmission is more prevalent in areas without basic sanitation (water treatment and sewage networks), which is a reality in low-income areas of Brazil. Intraspecies and interspecies contact can contribute to the spread of giardiasis (Feng and Xiao, 2011), a reality in low-income regions where cats and dogs can freely roam and livestock might be present. These multiple sources can increase the risk of infection, along with reinfection by *G. lamblia* and may explain the high infection prevalence observed in the present study. However, it was not known whether *Giardia* assemblage profile is associated with the infection burden.

Here, the three assemblages of *G. lamblia* (A, B, and E) that have been reported in humans were identified. A high rate of convergence between the gene targets (*gdh* and β*-gia*) used for genotyping was observed in our study. As the genes used for genotyping are considered conserved, a good discriminatory potential among the assemblages is expected (Ryan and Cacciò, 2013). However, the low nucleotide variability observed among subassemblages A questions the efficiency of these gene targets to characterize isolates in AI and AII, mainly (Lebbad et al., 2010, 2011; Ankarklev et al., 2018). Few nucleotide changes could be insufficient or sufficient to differentiation into subassemblages. Using the detection method to identify possible unique events recombination, the β*-gia* gene proved to be more conserved than the *gdh* gene. The high rate of recombination observed in assemblage A of the *gdh* gene points to high variability among isolates of this assemblage in this study. This justifies the divergence of characterization between the subassemblages AI and AII in the genes target. Events of recombination between subassemblages AI and AII and between assemblages A and E could occur (Xu et al., 2012; Ankarklev et al., 2018). We detected four cases of disagreement between assemblages A and E, but no recombination events were observed in this study. This disagreement among assemblages is commonly reported (Cacciò et al., 2008).

The frequency of assemblage A was higher in comparison to B or E. The few reports addressing the *Giardia* assemblages in Brazil point to regional differences in the prevalence of the predominant circulating assemblage (Coelho et al., 2017). In Rio de Janeiro, the assemblage A has been most frequently identified in humans (Volotão et al., 2007; Fantinatti et al., 2016), although assemblage B (Faria et al., 2016) and assemblage E (Fantinatti et al., 2016) have already been reported. The present results are consistent and reinforce the reported epidemiological profile of the *Giardia* assemblages in our region.

In our previous study conducted in this region, assemblage E and especially assemblage A were identified (Fantinatti et al., 2016). The results here show that the circulation of these assemblages is still current, with assemblage A being amplified throughout the environment by pets (Volotão et al., 2007; Fantinatti et al., 2018). However, the identification of assemblage B circulating in infected children had not been verified, although the first identification of assemblage B in Rio de Janeiro was reported in the same period (Faria et al., 2016). Further studies of environmental samples could help to understand the predominance of assemblage A.

In Brazil, the drug most often used by the public health service for the treatment of giardiasis is metronidazole and, in most cases, treatment is effective. However, the actual therapeutic failure rate of metronidazole in Brazil is still unknown. Our observed rate of continued infection after metronidazole treatment was considered high as 19 out of 36 re-evaluated children were infected at the first cure control. This continued after the second cure control where 15 out of 18 were positive. The positive cases in cure controls may be the result of therapeutic failure (drug subdose, parasite resistance) or reinfection (Argüello-García et al., 2004).

The negative results after metronidazole treatment are most probably a therapeutic success. This event was observed in individuals infected by the three different assemblages. It has been suggested that a determined assemblage may be associated with prolonged giardiasis and cases of persistence of infection after treatment (Mørch et al., 2008). No isolates from cure control samples were genotyped as assemblage B or E. This can suggest that treatment was effective for elimination of these assemblages. However, it is not possible to assert that these assemblages (B and E) are more sensitive to MTZ action than assemblage A. Since gene cloning was not performed in this study, it is not possible to rule out coinfection and only one assemblage was detected by the SANGER sequencing reactions.

As the studied community presents poor housing conditions and poor basic sanitation conditions, the main hypothesis is that cases of persistent infections are the result of successive infections. This is supported by those instances where the assemblage identified after a round of treatment was different from the first evaluation. Assemblage A was the only assemblage identified at the 1st and 2nd cure control. Considering the high frequency of assemblage A in our study area, it is most probable that reinfection would be by this assemblage. Except for three samples, 100% identity was observed between the sequences evaluated before and after in the same individual. Although the reinfection by the same assemblage cannot rule out in a short period, in our study area, in the reinfection events were expected about half of children infected by assemblages B or E in cure control (according to the first examination). This suggest the therapeutic failure and resistance should be considered.

As mentioned above, three individuals who persisted positive for assemblage A after a cycle of treatment presented a genotypic divergence between the first sample analyzed and the first cure control. The nucleotide changes were probably not a consequence of the treatment since these targets were not modified when *Giardia* trophozoites were *in vitro* exposed to different concentrations of MTZ. This suggests that these individuals were infected by another strain of the same assemblage but with a different subtype.

The findings in our study area suggest that children remain long periods infected with *G. lamblia*. The frequent episodes of reinfection and parasite persistence were remarkable findings in our study area. In areas with a high frequency of infection and reinfection, frequent use of metronidazole is also expected. However, it is not yet possible to define whether the recurrent cases were related to parasite drug resistance. Prolonged *Giardia* parasite infection can cause a reduction in fat-soluble vitamins and steatorrhea as well as stunting. The impact of giardiasis on child development reinforces the seriousness of this type of infection as a public health problem that, while mostly invisible and out of the public attention, requires greater care. The present results raise the question for areas with a high frequency of infection, could the treatment of giardiasis be exposing children to additional adverse effects from the drug while concomitantly masking the need for parasite control measures that require changes in public policies to encourage investments for improving sanitation conditions.

## Data Availability Statement

The datasets presented in this study can be found in online repositories. The names of the repository/repositories and accession number(s) can be found in the article/ Supplementary Material.

## Ethics Statement

The studies involving human participants were reviewed and approved by Comitê de Ética em Pesquisa do Instituto Oswaldo Cruz (CEP FIOCRUZ/IOC). Written informed consent to participate in this study was provided by the participants’ legal guardian/next of kin.

## Author Contributions

MF: conceptualization, methodology, funding acquisition, and manuscript writing. LL-O: methodology. TC-F: methodology. PA-T: methodology. EV: methodology. AB: formal analysis. AD-C: conceptualization, funding acquisition, and manuscript writing. All authors contributed to the article and approved the submitted version.

## Conflict of Interest

The authors declare that the research was conducted in the absence of any commercial or financial relationships that could be construed as a potential conflict of interest.

## Footnotes

1 http://doua.prabi.fr/software/cap3

2 www.grafpad.com/scientific-software/prism
