# Supplementary material for: Recirculation of Giardia lamblia Assemblage A After Metronidazole Treatment in an Area With Assemblages A, B, and E Sympatric Circulation
Source: Front Microbiol. 2020 Oct 22;11:571104. doi: 10.3389/fmicb.2020.571104 (PMC7642054; doi:10.3389/fmicb.2020.571104)
Supplement: Supplementary file 1 [file Data_Sheet_1.DOCX]

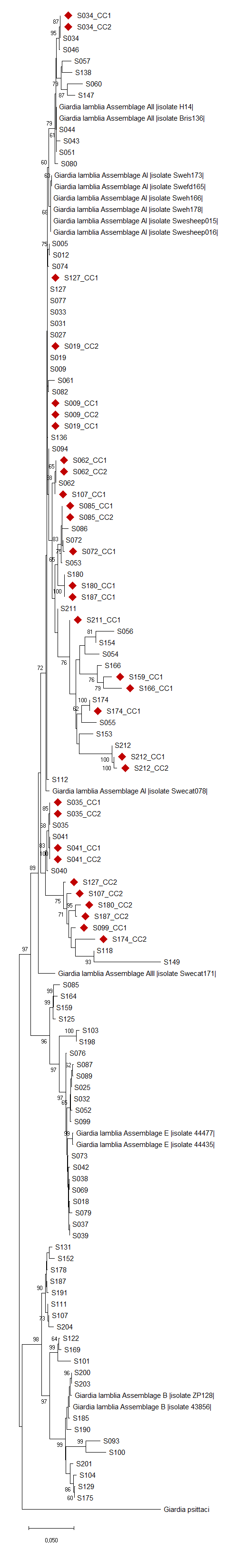


Supplementary Figure. Phylogenetic tree of the *Giardia lamblia* isolates from the children before and after treatment using concatenated *glutamate dehydrogenase* and *beta-giardin* sequences. The bootstrap values above 60% are shown at each branch. The isolate name was used to differentiate samples. Red diamond: CC1: isolate from a sample of first cure control, and CC2: isolate from a sample of second cure control.
