# Supplementary material for: Recirculation of Giardia lamblia Assemblage A After Metronidazole Treatment in an Area With Assemblages A, B, and E Sympatric Circulation
Source: Front Microbiol. 2020 Oct 22;11:571104. doi: 10.3389/fmicb.2020.571104 (PMC7642054; doi:10.3389/fmicb.2020.571104)
Supplement: Supplementary file 2 [file Table_1.DOCX]

Supplementary Table 1. Demographic and parasitological data of the children followed up in the study. M: male; F: female; NO: sample requested but not obtained; Hatched area: data not obtained.

| **Sample identification** | **Gender** | **Age in months** | **Diagnosis from the first exam** | **Assemblage** | **First cure control** | **Assemblage** | **Second cure control** | **Assemblage** |
| --- | --- | --- | --- | --- | --- | --- | --- | --- |
| 1 | M | 36,9 | *Ascaris lumbricoides* |  | NO |  |  |  |
| 2 | F | 22,1 | NEGATIVE |  |  |  |  |  |
| 3 | F | 32,0 | NEGATIVE |  |  |  |  |  |
| 4 | F | 20,7 | NEGATIVE |  |  |  |  |  |
| 5 | F | 42,2 | *Giardia lamblia* | A | NEGATIVE |  |  |  |
| 6 | F | 12,7 | NEGATIVE |  |  |  |  |  |
| 7 | M | 22,4 | NEGATIVE |  |  |  |  |  |
| 8 | F | 51,3 | NEGATIVE |  |  |  |  |  |
| 9 | M | 37,4 | *Giardia lamblia* | A | *Giardia lamblia* | A | *Giardia lamblia* | A |
| 10 | M | 33,9 | NEGATIVE |  |  |  |  |  |
| 11 | M | 47,4 | *Entamoeba histolytica/dispar* |  | NO |  |  |  |
| 12 | M | 40,9 | *Giardia lamblia* | A | NEGATIVE |  |  |  |
| 13 | M | 29,3 | *Ascaris lumbricoides* |  | NEGATIVE |  |  |  |
| 14 | M | 38,0 | NEGATIVE |  |  |  |  |  |
| 15 | M | 40,5 | NEGATIVE |  |  |  |  |  |
| 16 | M | 37,4 | NEGATIVE |  |  |  |  |  |
| 17 | M | 51,9 | NEGATIVE |  |  |  |  |  |
| 18 | M | 44,3 | *Giardia lamblia* | E | NO |  |  |  |
| 19 | M | 18,2 | *Giardia lamblia* | A | *Giardia lamblia* | A | *Giardia lamblia* | A |
| 20 | F | 35,6 | NEGATIVE |  |  |  |  |  |
| 21 | M | 30,4 | NEGATIVE |  |  |  |  |  |
| 22 | F | 51,8 | NEGATIVE |  |  |  |  |  |
| 23 | F | 41,1 | NEGATIVE |  |  |  |  |  |
| 24 | F | 54,1 | NEGATIVE |  |  |  |  |  |
| 25 | F | 28,0 | *Giardia lamblia* | E | NEGATIVE |  |  |  |
| 26 | M | 45,2 | NEGATIVE |  |  |  |  |  |
| 27 | M | 22,0 | *Giardia lamblia* | A | *Giardia lamblia* | NI | NO |  |
| 28 | F | 37,3 | NEGATIVE |  |  |  |  |  |
| 29 | F | 38,6 | NEGATIVE |  |  |  |  |  |
| 30 | M | 45,7 | *Endolimax nana* |  | NEGATIVE |  |  |  |
| 31 | M | 30,9 | *Giardia lamblia* | A |  |  |  |  |
| 32 | F | 30,9 | *Giardia lamblia* | E | NEGATIVE |  |  |  |
| 33 | F | 25,3 | *Giardia lamblia* | A | NO |  |  |  |
| 34 | M | 34,5 | *Giardia lamblia* | A | *Giardia lamblia* | A | *Giardia lamblia* | A |
| 35 | M | 26,4 | *Giardia lamblia* | A | *Giardia lamblia* | A | *Giardia lamblia* | A |
| 36 | F | 50,8 | *Endolimax nana* |  | NO |  |  |  |
| 37 | F | 22,6 | *Giardia lamblia* | E | NEGATIVE |  |  |  |
| 38 | F | 43,2 | *Giardia lamblia* | E | NO |  |  |  |
| 39 | F | 31,7 | *Giardia lamblia* | E | NEGATIVE |  |  |  |
| 40 | F | 23,9 | *Giardia lamblia* | A | NO |  |  |  |
| 41 | F | 41,7 | *Giardia lamblia* | A | *Giardia lamblia* | A | *Giardia lamblia* | A |
| 42 | M | 40,2 | *Giardia lamblia* | E | NEGATIVE |  |  |  |
| 43 | F | 36,1 | *Giardia lamblia* | A | NO |  |  |  |
| 44 | F | 45,4 | *Giardia lamblia* | A | NEGATIVE |  |  |  |
| 45 | M | 54,3 | *Entamoeba* *histolytica/dispar*  *Ascaris lumbricoides* |  | NO |  |  |  |
| 46 | F | 30,1 | *Giardia lamblia* | A | NO |  |  |  |
| 47 | F | 63,7 | *Endolimax nana* |  | NO |  |  |  |
| 48 | F | 33,6 | *Ascaris lumbricoides* |  | NO |  |  |  |
| 49 | F | 36,8 | *Ascaris lumbricoides*  *Endolimax nana* |  | NO |  |  |  |
| 50 | M | 22,5 | NEGATIVE |  |  |  |  |  |
| 51 | M | 49,7 | *Giardia lamblia* | A | NO |  |  |  |
| 52 | F | 33,6 | *Giardia lamblia* | E | NO |  |  |  |
| 53 | M | 38,5 | *Giardia lamblia* | A | NEGATIVE |  |  |  |
| 54 | M | 31,0 | *Giardia lamblia* | A | NO |  |  |  |
| 55 | F | 51,7 | *Giardia lamblia* | A | NO |  |  |  |
| 56 | F | 21,3 | *Giardia lamblia* | A/E | NO |  |  |  |
| 57 | F | 58,9 | *Giardia lamblia* | A | NO |  |  |  |
| 58 | F | 25,9 | NEGATIVE |  |  |  |  |  |
| 59 | F | 44,7 | NEGATIVE |  |  |  |  |  |
| 60 | M | 64,5 | *Giardia lamblia*  *Ascaris lumbricoides* | A | NO |  |  |  |
| 61 | M | 29,2 | *Giardia lamblia* | A | NO |  |  |  |
| 62 | M | 51,5 | *Giardia lamblia* | A | *Giardia lamblia* | A | *Giardia lamblia* | A |
| 63 | M | 50,5 | NEGATIVE |  |  |  |  |  |
| 64 | F | 25,4 | NEGATIVE |  |  |  |  |  |
| 65 | F | 38,0 | *Entamoeba* *histolytica/dispar*  *Ascaris lumbricoides* |  | NEGATIVE |  |  |  |
| 66 | F | 21,1 | NEGATIVE |  |  |  |  |  |
| 67 | F | 47,5 | NEGATIVE |  |  |  |  |  |
| 68 | F | 25,3 | NEGATIVE |  |  |  |  |  |
| 69 | M | 30,1 | *Giardia lamblia* | E | NO |  |  |  |
| 70 | M | 50,6 | NEGATIVE |  |  |  |  |  |
| 71 | M | 22,7 | NEGATIVE |  |  |  |  |  |
| 72 | M | 33,2 | *Ascaris lumbricoides*  *Giardia lamblia* | A | *Giardia lamblia* | A | *Giardia lamblia* | NI |
| 73 | F | 40,4 | *Giardia lamblia* | E | NO |  |  |  |
| 74 | F | 28,8 | *Giardia lamblia* | A | NO |  |  |  |
| 75 | F | 21,9 | NEGATIVE |  |  |  |  |  |
| 76 | F | 29,5 | *Giardia lamblia* | E | NO |  |  |  |
| 77 | F | 40,1 | *Giardia lamblia* | A | NO |  |  |  |
| 78 | F | 18,7 | NEGATIVE |  |  |  |  |  |
| 79 | F | 45,9 | *Giardia lamblia* | E | NO |  |  |  |
| 80 | M | 25,8 | *Giardia lamblia* | A | NO |  |  |  |
| 81 | F | 47,4 | NEGATIVE |  |  |  |  |  |
| 82 | F | 21,3 | *Giardia lamblia* | A | NO |  |  |  |
| 83 | M | 35,5 | NEGATIVE |  |  |  |  |  |
| 84 | M | 39,5 | NEGATIVE |  |  |  |  |  |
| 85 | F | 31,0 | *Giardia lamblia* | A/E | *Giardia lamblia* | A | *Giardia lamblia* | A |
| 86 | F | 40,8 | *Giardia lamblia* | A | NO |  |  |  |
| 87 | F | 42,1 | *Giardia lamblia* | E | NO |  |  |  |
| 88 | F | 42,8 | *Ascaris lumbricoides* |  | *Ascaris lumbricoides* |  |  |  |
| 89 | M | 26,9 | *Giardia lamblia* | E | NEGATIVE |  |  |  |
| 90 | M | 23,9 | *Endolimax nana* |  |  |  |  |  |
| 92 | M | 48,9 | NEGATIVE |  |  |  |  |  |
| 93 | F | 34,1 | *Giardia lamblia* | B | NO |  |  |  |
| 94 | F | 28,1 | *Giardia lamblia*  *Ascaris lumbricoides* | A | NEGATIVE |  |  |  |
| 96 | F | 23,7 | NEGATIVE |  |  |  |  |  |
| 98 | F | 34,9 | NEGATIVE |  |  |  |  |  |
| 99 | F | 25,0 | *Giardia lamblia* | E | *Giardia lamblia* | A | NEGATIVE |  |
| 100 | F | 29,6 | *Giardia lamblia* | B | NO |  |  |  |
| 101 | F | 32,7 | *Giardia lamblia* | B | NO |  |  |  |
| 102 | F | 54,2 | NEGATIVE |  |  |  |  |  |
| 103 | F | 36,7 | *Giardia lamblia* | E | NO |  |  |  |
| 104 | F | 34,0 | *Giardia lamblia* | B | NO |  |  |  |
| 105 | F | 23,0 | NEGATIVE |  |  |  |  |  |
| 106 | F | 32,3 | NEGATIVE |  |  |  |  |  |
| 107 | M | 34,4 | *Giardia lamblia* | B | *Giardia lamblia* | A | *Giardia lamblia* | A |
| 108 | M | 38,4 | NEGATIVE |  |  |  |  |  |
| 109 | F | 30,7 | NEGATIVE |  |  |  |  |  |
| 110 | F | 52,8 | *Entamoeba coli* |  | NO |  |  |  |
| 111 | M | 46,1 | *Giardia lamblia* | B | NEGATIVE |  |  |  |
| 112 | F | 27,3 | *Giardia lamblia*  *Entamoeba coli*  *Ascaris lumbricoides* | A | NO |  |  |  |
| 114 | M | 27,3 | NEGATIVE |  |  |  |  |  |
| 116 | M | 52,9 | NEGATIVE |  |  |  |  |  |
| 117 | M | 30,8 | NEGATIVE |  |  |  |  |  |
| 118 | M | 20,2 | *Giardia lamblia* | A | NO |  |  |  |
| 119 | M | 34,3 | *Entamoeba coli*  *Ascaris lumbricoides* |  | NO |  |  |  |
| 120 | M | 39,7 | NEGATIVE |  |  |  |  |  |
| 121 | M | 41,3 | NEGATIVE |  |  |  |  |  |
| 122 | M | 52,5 | *Giardia lamblia* | B | NO |  |  |  |
| 124 | F | 38,1 | NEGATIVE |  |  |  |  |  |
| 125 | F | 29,0 | *Giardia lamblia* | E | NEGATIVE |  |  |  |
| 126 | M | 21,1 | NEGATIVE |  |  |  |  |  |
| 127 | F | 20,2 | *Giardia lamblia* | A | *Giardia lamblia*  *Entamoeba histolytica/dispar* | A | *Giardia lamblia* | A |
| 128 | M | 30,2 | *Ascaris lumbricoides* |  | NO |  |  |  |
| 129 | F | 47,6 | *Giardia lamblia* | B | NO |  |  |  |
| 130 | F | 45,7 | NEGATIVE |  |  |  |  |  |
| 131 | M | 42,4 | *Giardia lamblia* | B | NO |  |  |  |
| 133 | F | 35,4 | NEGATIVE |  |  |  |  |  |
| 134 | F | 32,7 | NEGATIVE |  |  |  |  |  |
| 135 | M | 35,9 | NEGATIVE |  |  |  |  |  |
| 136 | F | 28,4 | *Giardia lamblia*  *Entamoeba coli* | A | NO |  |  |  |
| 137 | M | 30,5 | NEGATIVE |  |  |  |  |  |
| 138 | M | 34,0 | *Giardia lamblia* | A | NO |  |  |  |
| 139 | F | 44,0 | NEGATIVE |  |  |  |  |  |
| 141 | F | 50,6 | NEGATIVE |  |  |  |  |  |
| 143 | M | 46,7 | NEGATIVE |  |  |  |  |  |
| 144 | M | 27,1 | NEGATIVE |  |  |  |  |  |
| 145 | F | 42,9 | NEGATIVE |  |  |  |  |  |
| 146 | M | 43,7 | *Entamoeba histolytica/dispar*  *Endolimax nana*  *Ascaris lumbricoides* |  | NO |  |  |  |
| 147 | M | 38,4 | *Giardia lamblia* | A | NEGATIVE |  |  |  |
| 148 | M | 27,9 | NEGATIVE |  |  |  |  |  |
| 149 | F | 32,3 | *Giardia lamblia* | A | NO |  |  |  |
| 151 | F | 39,3 | NEGATIVE |  |  |  |  |  |
| 152 | F | 35,9 | *Giardia lamblia* | B | NO |  |  |  |
| 153 | F | 53,8 | *Giardia lamblia*  *Endolimax nana* | A | NO |  |  |  |
| 154 | F | 34,6 | *Giardia lamblia*  *Ascaris lumbricoides* | A | NO |  |  |  |
| 155 | M | 52,2 | NEGATIVE |  |  |  |  |  |
| 156 | M | 21,0 | NEGATIVE |  |  |  |  |  |
| 157 | F | 48,1 | NEGATIVE |  |  |  |  |  |
| 159 | M | 20,8 | *Giardia lamblia* | E | *Giardia lamblia*  *Endolimax nana* | A | *Giardia lamblia* | NI |
| 160 | M | 38,3 | NEGATIVE |  |  |  |  |  |
| 161 | M | 31,7 | NEGATIVE |  |  |  |  |  |
| 162 | F | 20,7 | NEGATIVE |  |  |  |  |  |
| 163 | F | 36,9 | NEGATIVE |  |  |  |  |  |
| 164 | F | 37,2 | *Giardia lamblia* | E | NO |  |  |  |
| 166 | F | 21,9 | *Giardia lamblia*  *Endolimax nana* | A | *Giardia lamblia* | A | NEGATIVE |  |
| 167 | F | 48,8 | NEGATIVE |  |  |  |  |  |
| 169 | M | 21,8 | *Giardia lamblia* | B | NEGATIVE |  |  |  |
| 170 | F | 45,6 | NEGATIVE |  |  |  |  |  |
| 171 | F | 27,2 | NEGATIVE |  |  |  |  |  |
| 173 | M | 21,6 | NEGATIVE |  |  |  |  |  |
| 174 | M | 28,2 | *Giardia lamblia* | A/E | *Giardia lamblia* | A | *Giardia lamblia* | A |
| 175 | F | 33,3 | *Giardia lamblia* | B | NO |  |  |  |
| 176 | F | 27,7 | NEGATIVE |  |  |  |  |  |
| 177 | M | 22,4 | *Endolimax nana* |  |  |  |  |  |
| 178 | F | 37,9 | *Giardia lamblia* | B | NO |  |  |  |
| 180 | F | 20,2 | *Giardia lamblia* | A | *Giardia lamblia*  *Endolimax nana* | A | *Giardia lamblia* | A |
| 181 | M | 41,8 | NEGATIVE |  |  |  |  |  |
| 182 | M | 41,2 | *Endolimax nana* |  | NO |  |  |  |
| 183 | F | 37,4 | *Endolimax nana* |  | NEGATIVE |  |  |  |
| 185 | F | 50,0 | *Giardia lamblia* | B | NO |  |  |  |
| 186 | F | 37,3 | NEGATIVE |  |  |  |  |  |
| 187 | M | 27,9 | *Giardia lamblia*  *Endolimax nana* | B | *Giardia lamblia* | A | *Giardia lamblia* | A |
| 188 | M | 42,6 | NEGATIVE |  |  |  |  |  |
| 189 | M | 24,6 | NEGATIVE |  |  |  |  |  |
| 190 | M | 42,0 | *Giardia lamblia* | B | NO |  |  |  |
| 191 | M | 34,7 | *Giardia lamblia* | B | NO |  |  |  |
| 192 | F | 25,4 | NEGATIVE |  |  |  |  |  |
| 193 | M | 45,2 | NEGATIVE |  |  |  |  |  |
| 195 | F | 33,9 | NEGATIVE |  |  |  |  |  |
| 196 | F | 21,7 | NEGATIVE |  |  |  |  |  |
| 198 | F | 27,9 | *Giardia lamblia* | A/E | NEGATIVE |  |  |  |
| 199 | M | 29,2 | NEGATIVE |  |  |  |  |  |
| 200 | M | 35,2 | *Giardia lamblia* | B | NO |  |  |  |
| 201 | F | 34,5 | *Giardia lamblia* | B | NO |  |  |  |
| 202 | M | 37,7 | NEGATIVE |  |  |  |  |  |
| 203 | F | 33,3 | *Giardia lamblia* | B | NO |  |  |  |
| 204 | F | 27,9 | *Giardia lamblia* | B | NEGATIVE |  |  |  |
| 206 | F | 37,5 | *Entamoeba coli* |  | NO |  |  |  |
| 207 | M | 32,2 | NEGATIVE |  |  |  |  |  |
| 208 | F | 52,8 | NEGATIVE |  |  |  |  |  |
| 209 | F | 43,0 | NEGATIVE |  |  |  |  |  |
| 211 | M | 20,6 | *Giardia lamblia*  *Endolimax nana* | A | *Giardia lamblia*  *Endolimax nana* | A | NEGATIVE |  |
| 212 | M | 38,9 | *Giardia lamblia* | A | *Giardia lamblia* | A | *Giardia lamblia* | A |
| 213 | F | 33,9 | NEGATIVE |  |  |  |  |  |
| 214 | F | 21,7 | NEGATIVE |  |  |  |  |  |

M: male; F: female; NO: sample requested but not obtained. Hatched area: data not obtained.
