# Supplementary material for: Recirculation of Giardia lamblia Assemblage A After Metronidazole Treatment in an Area With Assemblages A, B, and E Sympatric Circulation
Source: Front Microbiol. 2020 Oct 22;11:571104. doi: 10.3389/fmicb.2020.571104 (PMC7642054; doi:10.3389/fmicb.2020.571104)
Supplement: Supplementary file 3 [file Table_2.DOCX]

Supplementary Table 2. *Giardia lamblia* sequences obtained from GenBank and used as references for the construction of *glutamate dehydrogenase* and *beta-giardin* genes phylogenetic trees.

| **Gene target** | **Isolate name** | **Acess number** | **Host** | **Country** | **Assemblage** | **Author** |
| --- | --- | --- | --- | --- | --- | --- |
| *glutamate dehydrogenase* | Swesheep015 | JF773750 | Sheep | Sweden | AI | Lebbad et al. |
|  | Sweh178 | GQ329676 | Human | Sweden | AI | Lebbad et al. |
|  | Swesheep016 | JF773751 | Sheep | Sweden | AI | Lebbad et al. |
|  | Sweh166 | GQ329674 | Human | Sweden | AI | Lebbad et al. |
|  | Sweh173 | GQ329675 | Human | Sweden | AI | Lebbad et al. |
|  | Swecat078 | JF773753 | Cat | Sweden | AI | Lebbad et al. |
|  | Swefd165 | JF773754 | *Dama dama* | Sweden | AI | Lebbad et al. |
|  | Ad-1 | AY178735 | Human | Australia | AI | Ey et al. |
|  | Send H-20 | KT948091 | Human | Ethiopia | A | Wegayehu et al. |
|  | H14 | KY658185 | Human | Uganda | A | Nolan et al. |
|  | Bris-136 | AY178737 | Human | Australia | AII | Ey et al. |
|  | Swecat171 | EU769223 | Cat | Sweden | AIII | Lebbad et al. |
|  | 43856 | MG746611 | Human | Egypt | B | Naguib et al. |
|  | ZP-128 | MK982528 | Rhesus macaque | Bangladesh | B | Karim, M.R. |
|  | 27 / 65 | KX085495 | Human | Brazil | B | Faria et al. |
|  | 44435 | MG820461 | Calf | Egypt | E | Naguib et al. |
|  | 44477 | MG820462 | Calf | Egypt | E | Naguib et al. |
|  | SG-20 | KC960649 | Sheep | China | E | Liu et al. |
| *beta-giardin* | Swesheep016 | JF773748 | Sheep | Sweden | AI | Lebbad et al. |
|  | Swesheep015 | JF773747 | Sheep | Sweden | AI | Lebbad et al. |
|  | Swefd165 | JF773749 | *Dama dama* | Sweden | AI | Lebbad et al. |
|  | Sweh166 | GQ329671 | Human | Sweden | AI | Lebbad et al. |
|  | Sweh173 | GQ329672 | Human | Sweden | AI | Lebbad et al. |
|  | Sweh178 | GQ329673 | Human | Sweden | AI | Lebbad et al. |
|  | Swecat078 | EU769205 | Cat | Sweden | AII | Lebbad et al. |
|  | H14 | KY658181 | Human | Uganda | A | Nolan et al. |
|  | Be2 | EU014384 | - | USA | A | Teodorovic et al. |
|  | VANC/85/UBC/2 | KM190682 | Human | Canada | A | Prystajecky et al. |
|  | Send H82 | KT948087 | Human | Ethiopia | A | Wegayehu et al. |
|  | Sweh040 | HM165227 | Human | Sweden | AII | Lebbad et al. |
|  | R2 | FJ560582 | Human | France | AII | Bonhomme et al. |
|  | Swecat171 | EU769206 | Cat | Sweden | AIII | Lebbad et al. |
|  | Swecattle | EU769214 | Cattle | Sweden | E | Lebbad et al. |
|  | ECUST21169 | KY769091 | Calf | China | E | Wang et al. |
|  | Chan C94 | KT922249 | Calf | Ethiopia | E | Wegayehu et al. |
|  | ECUST20773 | KY633470 | Yak | China | E | Jin et al. |
|  | ZP-128 | MK982526 | Rhesus macaque | Bangladesh | B | Karim |
|  | S24C3 | KU504732 | Human | Brazil | B | Coronato Nunes et al. |
|  | Sweh043 | HM165215 | Human | Sweden | B | Lebbad et al. |
|  | Sweh001 | HM165208 | Human | Sweden | B | Lebbad et al. |
|  | VANC/90/UBC/54 | KP687756 | Human | Canada | B | Prystajecky et al. |
|  | Hole H2 | KT948083 | Human | Ethipia | B | Wegayehu et al. |
|  | CBHRG13 | DQ116616 | Sheep | Mexico | E | Di Giovanni et al. |
